# Differential expression of NBS-LRR-encoding genes in the root transcriptomes of two *Solanum phureja* genotypes with contrasting resistance to *Globodera rostochiensis*

**DOI:** 10.1186/s12870-017-1193-1

**Published:** 2017-12-28

**Authors:** Alex V. Kochetov, Anastasiya Y. Glagoleva, Kseniya V. Strygina, Elena K. Khlestkina, Sophia V. Gerasimova, Salmaz M. Ibragimova, Natalja V. Shatskaya, Gennady V. Vasilyev, Dmitry A. Afonnikov, Nikolay A. Shmakov, Olga Y. Antonova, Tatyana A. Gavrilenko, Natalia V. Alpatyeva, Alexander Khiutti, Olga S. Afanasenko

**Affiliations:** 1 Institute of Cytology and Genetics, SB RAS, Novosibirsk, 630090 Russia; 2 Novosibirsk State University, Novosibirsk, 630090 Russia; 3 Vavilov Institute of Plant Genetic Resources (VIR), Saint Petersburg, 190000 Russia; 4 St. Petersburg State University, St. Petersburg, 199034 Russia; 5 All Russian Research Institute for Plant Protection, Saint Petersburg, 196608 Russia; 6 Novosibirsk State Agrarian University, Novosibirsk, 630039 Russia

**Keywords:** NBS-LRR genes, *Solanum phureja*, Resistance, *Globodera rostochiensis*

## Abstract

**Background:**

The characterization of major resistance genes (R genes) in the potato remains an important task for molecular breeding. However, R genes are rapidly evolving and frequently occur in genomes as clusters with complex structures, and their precise mapping and identification are complicated and time consuming.

**Results:**

Comparative analysis of root transcriptomes of *Solanum phureja* genotypes with contrasting resistance to *Globodera rostochiensis* revealed a number of differentially expressed genes. However, compiling a list of candidate R genes for further segregation analysis was hampered by their scarce annotation. Nevertheless, combination of transcriptomic analysis with data on predicted potato NBS-LRR-encoding genes considerably improved the quality of the results and provided a reasonable number of candidate genes that provide *S. phureja* with strong resistance to the potato golden cyst nematode.

**Conclusion:**

Combination of comparative analyses of tissue-specific transcriptomes in resistant and susceptible genotypes may be used as an approach for the rapid identification of candidate potato R genes for co-segregation analysis and may be used in parallel with more sophisticated studies based on genome resequencing.

**Electronic supplementary material:**

The online version of this article (10.1186/s12870-017-1193-1) contains supplementary material, which is available to authorized users.

## Background

New disease resistance genes (R genes) have been commonly introduced into crop plants through intra- and/or interspecific introgressive hybridization. Both cultivated and closely related wild species have been used for this purpose for a long time. Marker-assisted selection is efficiently exploited to facilitate the successful breeding of new resistant cultivars and to combine several R genes into a single genotype [1, 2]. Mapping resistance loci is commonly performed by phenotyping segregating populations and genotyping them with a large number of genetic markers. Some R genes have been cloned and characterized. It was revealed that nucleotide-binding site-leucine-rich repeat (NBS-LRR) genes compose the largest plant resistance gene family, accounting for ∼80% of more than 140 cloned R genes [3]. However, the search for R gene variants providing plant varieties with resistance against a specific pathogen or new pathogen races is still complicated and time consuming. In many cases, the responsible R gene remains unidentified, and genetic markers (if available) are associated with qualitative trait loci (QTLs) containing several (or many) candidate genes.

Recently, new approaches in this field were developed on the basis of genomic data (some recently published examples are presented below). Genome-wide resequencing and comparison with reference genomes revealed a number of NBS-LRR candidate genes in common wild rice [4], *Medicago truncatula* [5], *Arachis duranensis* and *A. hypogaea* [6]. Comparison of syntenic genomic regions of related species containing NBS-LRR genes was found to be a promising way to locate candidate resistance genes in the genomes of various crops (e.g., [7, 8]). Sometimes, R genes in resistant cultivars were not found in reference genomes; e.g., quantitative trait loci in Spanish barley landrace on the long arm of chromosome 7H provided resistance against powdery mildew and contained a cluster of NBS-LRR genes absent in the reference barley genome [9]. Many R gene analogs (RGAs) have conservative domains and may be predicted by bioinformatic tools [10] that facilitate their identification.

Combination of genomic and transcriptomic approaches provides an efficient way to identify candidate R genes for further verification. For example, a search for ascochyta blight resistance genes located close to nine QTLs in the chickpea genome revealed approximately 30 NBS-LRR candidate genes. Further comparison of their transcription patterns in resistant and susceptible genotypes revealed five candidate genes with genotype–specific expression [11]. The investigation of QTLs associated with willow resistance against leaf rust revealed a candidate TIR-NBS-LRR gene whose constitutive expression was considerably lower in the susceptible genotype before and after inoculation with *Melampsora larici-epitea* [12]. Comparative transcriptome analysis of *Gossypium hirsutum* genotypes resistant and susceptible to reniform nematodes revealed a number of candidate RGAs located close to quantitative trait loci [13]. RNA-seq of resistant recombinant inbred lines of *Arachis hypogaea* at different time points after inoculation with the nematode *Meloidogyne arenaria* revealed the molecular mechanisms of pathogenesis and plant defenses as well as a constitutively expressed TIR-NBS-LRR gene that potentially activates an effector-induced immune response [14].

Analysis of the genomes and transcriptomes of resistant plant genotypes commonly results in a list of candidate R genes that should be further tested by co-segregation analysis or other tools of reverse genetics. Various experimental approaches were developed to identify the candidate NBS-LRR genes responsible for the recognition of specific pathogens (effectoromics (defined as a high-throughput, functional genomics approach that uses effectors for probing the plant germplasm to detect R genes [15]), dsRNA-mediated suppression of a candidate gene in a resistant plant [16], overexpression of NBS-LRR genes in susceptible plants [17, 18], etc.). However, the search for NBS-LRR genes of interest is hampered by their natural variability; commonly, genomes of cultivated plants contain clusters with dozens of duplicated and reorganized RGAs with highly similar structures [19, 20].

One of the potential methods of rapid target gene identification may be the combination of comparative transcriptome analysis of resistant and susceptible plant genotypes with bioinformatic predictions of NBS-LRR-related transcripts (the predictions could be based on their conservative NBS domain (e.g., [21, 22]) or other computational techniques [23]). We applied this approach to evaluate the number of differentially expressed NBS-LRR genes on the model of root transcriptomes of two *Solanum phureja* accessions of different origins from the VIR collection. These accessions are likely to be characterized by different sets of evolved R genes, and it was found earlier that these genotypes were at least different in their resistance to the potato wart *Synchytrium endobioticum* [24] and to the golden potato cyst nematode *Globodera rostochiensis* (Wollenweber) Behrens (GPCN) [25]. The resistant genotype contains no genetic markers to the known GPCN strong resistance genes *Gro1–4* and *H1* [25] and possibly bears new R gene variants. We hypothesize that the usage of tissue-specific transcriptomes for the prediction of NBS-LRR-related transcripts results in the rapid identification of candidate R genes for further experimental verification.

Potato cyst nematodes originated in Andean regions of South America [26]. At present, GPCN is found worldwide and is one of the most economically important potato pathogens [27]. Currently, *G. rostochiensis* occurs locally in some regions of the European part of Russia, southern Siberia, and the Far East of Russia [25, 28]. Depending on the potato cultivar, yield losses can range from 19% to 90% [29], and GPCN eggs can remain dormant and viable within the cyst for 30 years [30]. Most chemical nematicides are not efficient [31, 32] or are prohibited in Europe, and the control of GPCN is mainly based on the deployment of single resistance genes (R-genes). However, only a few R genes are available, and their efficacy is threatened by the capacity of nematodes to evolve. R genes conferring strong resistance to the pathotype Ro1 of *G. rostochiensis* were introgressed into commercial potato varieties from Andean potato species: the *H1* gene from the cultivated species *Solanum tuberosum subsp. andigenum* [33] and the *Gro1–4* gene from the Bolivian wild species *S. spegazzinii* [34, 35]. Since the *S. phureja* genotypes used in this investigation contained no markers for *H1* and *Gro1–4* genes [25], the resistant genotype likely contains a new R gene variant. One of the aims of this study was to compile a set of new candidate R genes against GPCN for further investigation and inclusion in potato breeding programs or other biotechnological approaches for the improvement of plant resistance to pathogens (e.g., [18, 36–39]).

## Methods

### Plant material

Two accessions of diploid cultivated species *S. phureja* k-11,291 (collected in Peru) and k-9836 (from Bolivia) were selected from the VIR potato collection. Each accession was represented by one clone (genotype) with the VIR introduction numbers i-0144787 (k-11,291) and i-0144786 (k-9836), respectively. These accessions were characterized by nuclear SSRs, chromosome counts, and morphological features [40]. According to plastid SSRs data, these accessions have unequal haplotypes, indicating different maternal origins [41]. It was previously found that these genotypes differed in their resistance to GPCN (pathotype Ro1): i-0144786 is susceptible, whereas i-0144787 is highly resistant but contains no DNA markers of *Gro1–4* and *H1* (TG689, 239E4 left/Alu I, and Gro1–4) [25]. *S. tuberosum* cultivars ‘Nevsky’ and ‘Red Scarlett’ (susceptible and resistant to GPCN, respectively) were used as controls.

### Evaluation of *S. phureja* resistance to GPCN

A population of *G. rostochiensis* (pathotype Ro1) from an infested plot in the Leningrad Region, Russia (Belogorka), was characterized previously [25] with appropriate molecular markers [42]. The nematode population was propagated on the susceptible cultivar ‘Nevsky’ under greenhouse conditions. Cysts were extracted from soil by the flotation technique and stored for 4 months at 4 °C.

To stimulate root formation, potato tubers were placed on sterile watered sand in trays within 2 weeks, and each tuber was further transferred to 10-cm-diameter plastic pots (500 ml) half filled with sterile soil and used for inoculation by GPCN. Before inoculation, in order to estimate the nematode population densities, cysts were crushed, and the contents of nematode eggs and juveniles were calculated. Inoculation by GPCN was performed by spraying 1 ml of water suspension with approximately 1500 eggs and juveniles on the roots of one potato tuber. After inoculation, the tubers were covered with sterile soil, and plants were incubated at 4000 lx, 16 h of light, and 22 °C [25]. Infected roots, stained with acid fuchsin were scanned for the presence of nematodes under an AxioScope A1 light microscope (Carl Zeiss, Germany).

For evaluation of plant resistance to GPCN, cysts were extracted from the roots by the flotation technique 3 months after inoculation and crushed, and the numbers of juveniles and eggs were calculated. Then, using the following standard scoring system (OEPP/EPPO, 2006), the degree of resistance to GPCN was recorded: scores of 9–7, highly resistant; scores of 6–4, moderately resistant; and scores of 3–1, susceptible.

### RNA extraction

For RNA-seq, roots were collected 72 h after inoculation. For each genotype, three infected and three control (water-inoculated) plants were used. The roots of these plants were thoroughly rinsed with sterile distilled water, fixed in liquid nitrogen and used for RNA extraction. Total RNA was extracted with an RNeasy Plant Mini Kit (Qiagen).

### RNA-seq analysis

The quality of RNA samples was evaluated using a Bioanalyzer 2100 (Agilent). ERCC Spike-In Mix2 was added to each RNA sample prior to poly-A mRNA extraction using a Dynabeads mRNA Purification Kit (Ambion). RNA-seq library preparations were carried out using an Ion Total RNA-Seq Kit v2 (Life Technologies) according with the manufacturer’s instructions with modifications. Chemical 5-min-long RNA fragmentation was used instead of an enzymatic treatment to increase the reproducibility and proportion of long fragments. Size selection using Caliper LabChip XT (Perkin-Elmer) was carried out to obtain library inserts 250–300 bp long. E-PCR, enrichment and quantification for Ion Torrent sequencing were performed with One-Touch 2 and One-Touch ES systems (Life Technologies). Sequencing was carried out on the Ion PGM (Life Technologies) using Hi-Q View sequencing kits and 318v2 chips. ERCC analysis demonstrated the absence of significant misrepresentation (R-squared values, 0.93–0.97).

#### qRT-PCR

For qPCR, RNA was treated with DNAse (Qiagen RNase-Free DNase Set). A 0.7 μg aliquot of RNA was used to prepare single-stranded cDNA by reverse transcription based on a RevertAid™ kit (Thermo Fisher Scientific Inc., Waltham, MA, USA) and a (dT)_15_ primer.

Primers were designed using IDT PrimerQuest software (http://eu.idtdna.com/PrimerQuest/Home/) for ten DEGs.

The β-tubulin gene sequence (Accession number: 609,267) was used as a reference. The following primer sequences were designed using OLIGO software: Forward, 5`-AGCTTCTGGTGGACGTTATG-3`, and Reverse, 5`-ACCAAGTTATCAGGACGGAAGA-3`. The subsequent qRT-PCR was based on a SYNTOL SYBR Green I kit (Syntol, Moscow, Russia). Three technical replicates of each reaction were run.

### Bioinformatic analysis of RNA-seq data

#### Library preprocessing

The Prinseq tool [43] was used to assess sequence quality and filter the libraries. Nucleotide sequences larger than 50 nt and with a mean Phred quality score greater than 20 were used for further analysis.

#### Library mapping

We used the *S. tuberosum* group *Phureja* clone DM1–3516 R44 (genome version 3.0.34, European Nucleotide Archive ID GCA_000226075.1 [44] as a reference. Nucleotide sequences and their annotations were downloaded from the Ensembl Plants database [45]. In addition, the locations of 755 predicted NB-LRR loci [46] were mapped on the reference genome by aligning their sequences with the aid of the Gmap tool [47] (positions of potential R genes are listed in Additional file 1).

To map the filtered libraries in the genome, the TopHat2 [48] tool was implemented after constructing genome indexes with Bowtie2 software [49]. Read alignments were processed with the Cufflinks pipeline [50]. Numbers of read counts mapped to each genome segment, either expressed or annotated in the genome assembly (‘transcripts’), and corresponding RPKM (reads per kilobase per million mapped reads) values [51] were used to detect differentially expressed genes (DEGs) between the *S. phureja* accessions studied.

#### DEGs prediction

Analysis of differential expression of *S. phureja* genes was performed using Cuffdiff utility of Cufflinks pipeline. Transcripts with total RPKM values lesser than 12 were discarded. Transcript was considered differentially expressed in two libraries if it had two-fold or higher difference in abundance (|logFC| > 1, significance level q < 0.05). For functional analysis, up- and down-regulated transcripts were analyzed separately.

Data on characteristic peptides (peptide IDs) were taken from annotation in Spud database (http://solanaceae.plantbiology.msu.edu/data/PGSC_DM_v3.4_g2t2c2p2func_nonredundant.txt.zip) [52] that provides the links between the gene and corresponding transcripts, CDS and peptides. Lists of peptide IDs for significantly up- and down-regulated genes were processed with AgriGO database [53] to evaluate the enriched gene ontology terms for these DEGs.

## Results

### Verification of resistance levels of *S. phureja* accessions i-01444786 and i-01444787 to GPCN

**Fig. 1:**
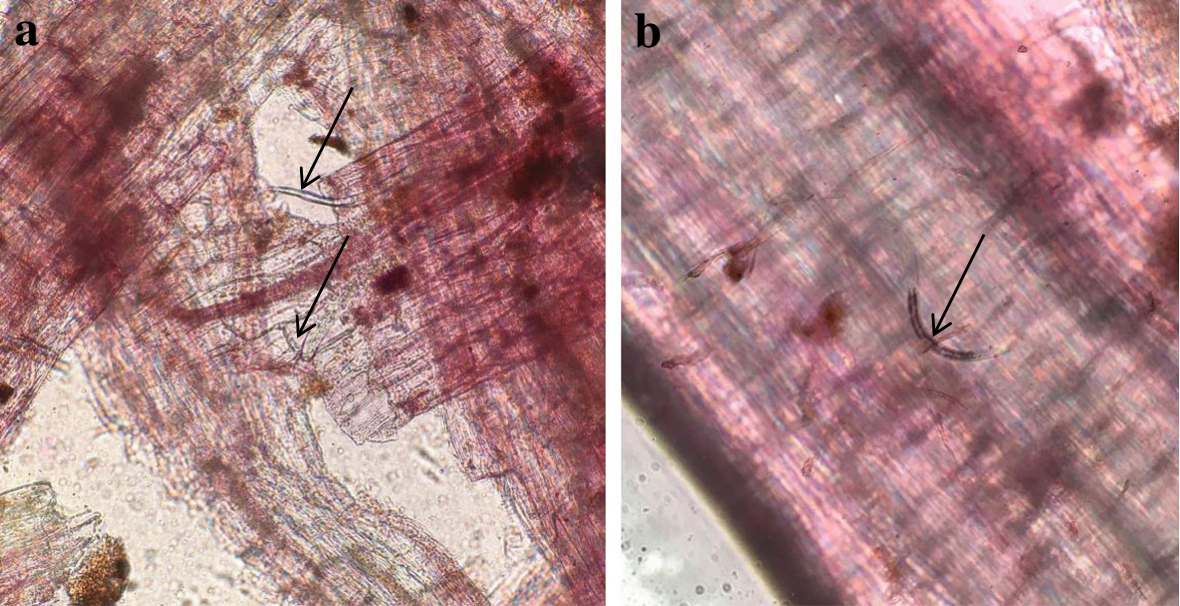
GPCN juvenile penetration into the root tissues of the susceptible *S. phureja* accession i-0144786 (**a**) and resistant *S. phureja* accession i-0144787 (**b**) (3 h after inoculation; arrows mark the juveniles)

**Fig. 2:**
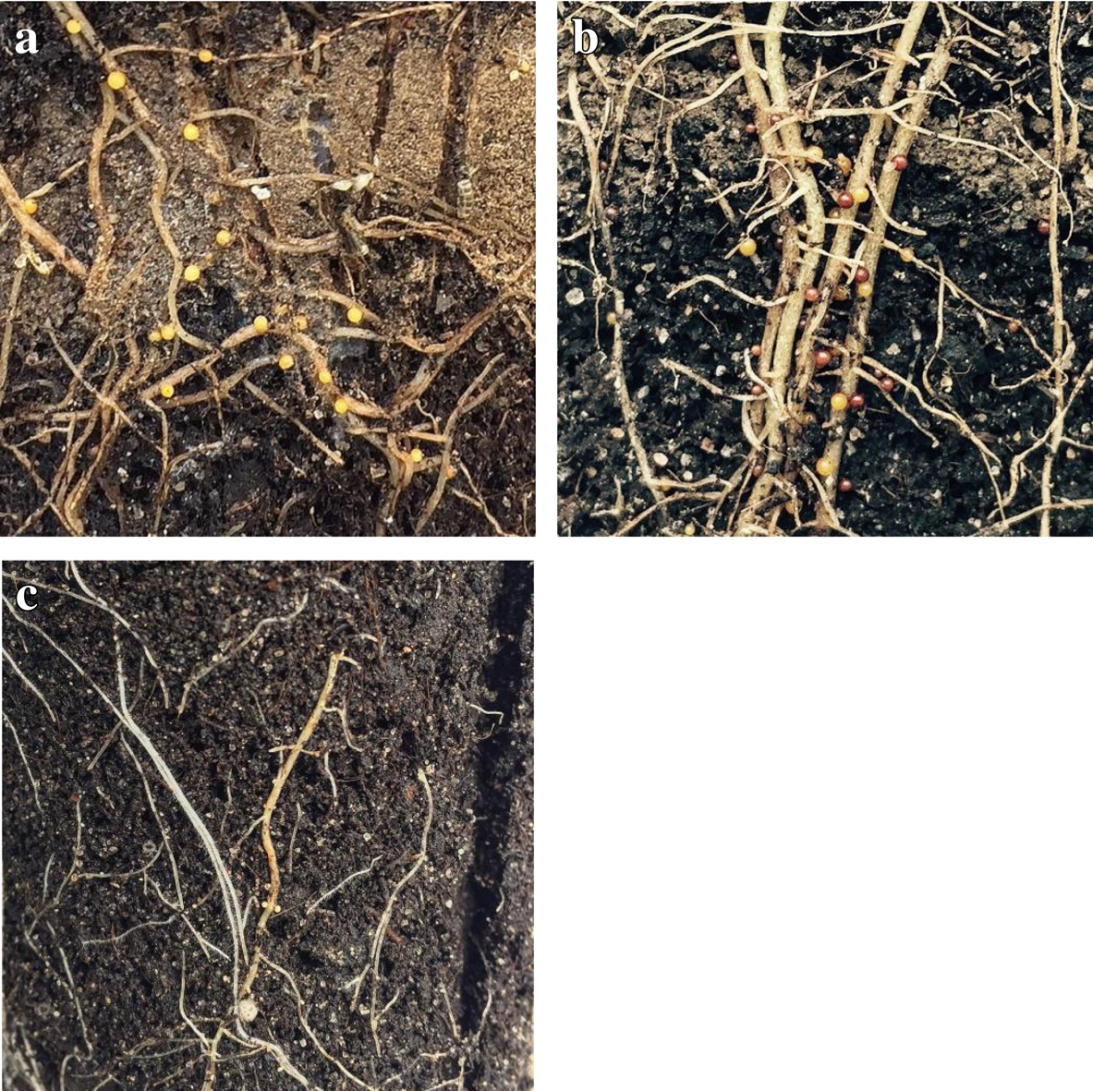
Images of roots with cysts of GPCN after 3 months of inoculation of the susceptible *S. phureja* accession i-0144786 (**a**), the susceptible *S. tuberosum* cultivar Nevsky (**b**), and the resistant *S. phureja* accession i-0144787 (**c**)

Roots of *S. phureja* accessions i-0144787, i-0144786, *S. tuberosum* susceptible cultivar ‘Nevsky’ (10 tubers) and resistant cultivar ‘Red Scarlett’ (10 tubers) were inoculated with GPCN and analyzed at several time points. Penetration of roots of both *S. phureja* genotypes by GPCN juveniles were detected starting from 3 h after inoculation (Fig. 1). It was detected that GPCN formed a large number of cysts after 3 months of cultivation on the roots of both *S. phureja* i-0144786 and susceptible control ‘Nevsky’ (Fig. 2) but not on the roots of *S. phureja* i-0144787 or the resistant *S. tuberosum* cultivar ‘Red Scarlett’ (Table 1). According to the international 9-score scale [44], *S. phureja* i-0144786 and cultivar ‘Nevsky’ were susceptible (scores of 2 and 1, respectively), whereas *S. phureja* i-0144787 and cultivar ‘Red Scarlett’ were resistant (scores of 7 and 9, respectively) (Table 1). These data confirmed the previously reported results (i-0144786, score of 2; i-0144787, scores of 7–9 [25]).

**Table 1:** Resistance of two *S. phureja* accessions to pathotype Ro1 *G. rostochiensis*

VIR catalog number	VIR introduction number	Characteristic of resistance	
		Score	Resistance
*S. phureja* 9836	i-0144786	2	S
*S. phureja* 11,291	i-0144787	7	R
cv. Nevsky (susceptible control)		1	S
cv. Red Scarlett (resistant control)		9	R

For transcriptome analysis, samples of roots of *S. phureja* accessions i-0144786 and i-0144787 were obtained after 72 h of inoculation with either nematode or water (pooled from 3 plants per library). In total, 12 samples were obtained (three technical replicates) for i-0144786/water (Sus_cont), i-0144787/water (Res_cont), i-0144786/GPCN (Sus_nem), and i-0144787/GPCN (Res_nem).

### Library preprocessing

Twelve libraries of pooled reads, containing a total of 48,059,222 short reads comprising 7.44 gigabases, were produced as raw sequencing data. Filtering resulted in 47,310,018 short reads that comprised 7.31 gigabases of sequences after the removal of 1.5% of short reads (Table 2).

**Table 2:** Metrics of *S. phureja* short-read sequenced libraries^a^

Library	Clean reads, mln	Mean length, nucl.	Mapped reads, %
Sus_cont0	1.28	164	52.6
Sus_cont1	5.9	140	61.2
Sus_cont2	4.48	153	64.0
Sus_nem0	5.29	169	39.7
Sus_nem1	3.16	152	52.0
Sus_nem2	4.57	159	55.7
Res_cont0	4.6	160	58.1
Res_cont1	2.98	152	54.0
Res_cont2	3.86	169	56.9
Res_nem0	4.15	143	51.2
Res_nem1	4.05	162	41.5
Res_nem2	2.89	127	50.8

^a^Sus_cont - i-0144786/water; Sus_nem - i-0144786/GPCN; Res_cont - i-0144787/water; Res_nem - i-0144787/GPCN; 0, 1, and 2 – the number of technical replicates

### Differential gene expression in the roots of resistant and susceptible *S. phureja* genotypes

Analysis of *S. phureja* transcriptomic data with the aid of the Cufflinks pipeline revealed 45,171 genome fragments corresponding to both annotated genes and unannotated genome segments in the reference genome assembly of *S. tuberosum* [45]. RPKM values were counted, and the amounts of DEGs in *S. phureja* accessions i-0144786 and i-0144787 are listed in Table 3 (a detailed description is available in Additional file 2).

**Table 3:** Numbers of DEGs between the root transcriptomes of *S. phureja* genotypes resistant (Res) or susceptible (Sus) to GPCN infection (cont – inoculation with water; nem – inoculation with GPCN)

DEGs	Sus_cont vs Res_cont	Sus_nem vs Sus_cont	Res_nem vs Res_cont	Sus_nem vs Res_nem
Up-regulated	638	234	353	285
Down-regulated	463	203	253	180

To verify the RNA-seq results, transcripts of 10 DEGs that were more abundant in the *S. phureja*-resistant genotype transcriptomes were selected. The log_2_(FC) values predicted by RNA-seq data and the experimental log_2_(FC) values for verified genes as well as the sequences of primers and other technical information are shown in Additional file 3. The NBS-LRR-encoding genes were preferentially used for verification. This list included *S. phureja* DEGs similar to the following genes from the reference genome: late blight resistance protein Rpi-blb2 (PGSC0003DMG400004561), TMV resistance protein N (PGSC0003DMG400020722), Tospovirus resistance protein C (PGSC0003DMG402016602), Rpi protein (PGSC0003DMG400023288), late blight resistance protein (PGSC0003DMG400005970), Cc-nbs-lrr resistance protein (PGSC0003DMG400026666), disease resistance protein R3a (PGSC0003DMG402027402), disease resistance protein (PGSC0003DMG400018464), Nbs-lrr resistance protein (PGSC0003DMG400013308), and HJTR2GH1 protein (PGSC0003DMG400011517). The results of the qPCR supported the RNA-seq data. In all cases, the target transcripts were more abundant in transcriptomes of resistant genotypes, and in 8 cases, the difference was larger than twofold and statistically significant (Additional file 4).

Since these DEGs were revealed by the alignment to the annotated reference potato genome, we carried out a Gene Ontology term search for genes up- and down-regulated in the GPCN-resistant genotypes. For down-regulated genes, the enriched GO terms included ‘translation’ (*p* = 0.0011), ‘nucleosome assembly’ (*p* = 9.17·10^−4^), ‘nucleosome’ (*p* = 5.6·10^−4^) and ‘structural constituent of ribosome’ (*p* = 2.4·10^−4^). Since the inoculation of plant roots with either water or nematode resulted in tissue wounding, the inhibition of the expression of house-keeping genes likely reflects the response to this stressful condition (Additional file 5). For up-regulated genes, the most enriched GO terms included ‘response to oxidative stress’ (*p* = 5.72·10^−16^) and ‘peroxidase activity’ (*p* = 3.05·10^−16^) (Additional file 6). These terms reflect the non-specific cellular responses to stressful conditions commonly resulting in generation of ROS (reactive oxygen species) and oxidative stress (sometimes followed by programmed cell death as a hypersensitive response), as well as the synthesis of peroxidases for cell wall modification. In general, GO term enrichments corresponded to the expected transcriptome reprogramming in the frame of a combined non-specific response to the root wounding and the onset of a specific response to the GPCN infestation (72 h after inoculation).

Closer inspection of the DEG list revealed a remarkable difference between the genotypes. One may see that the transcriptomes of the resistant *S. phureja* genotype is characterized by higher content of the transcripts similar to various potato defense-related genes according to their annotation ([52, 54] ‘description’ field) (Additional file 2). Since the aim of this study concerns the identification of major R genes providing strong resistance to GPCN, the most probable candidates are likely to belong to the NBS-LRR family. However, annotation of *S. tuberosum* genes is frequently scarce, and only a very few DEGs revealed in this study contained the specific term ‘NBS-LRR’ or a similar term in the ‘description’ field, whereas non-specific terms such as ‘disease resistance’ were more abundant (Additional file 2). Thus, we used additional information on 755 NB-LRR loci predicted in the potato genome [46]. This analysis revealed approximately 330 *S. phureja* root transcripts potentially coding for NBS-LRR related proteins (Additional file 7). This list of DEGs was ranked on the basis of the following simple description (Additional file 8): Group 1 contained the most probable candidate genes with either no or very little mRNA representation in roots of the susceptible *S. phureja* genotype but represented in the roots of the resistant i-0144787 genotype (2 genes). Group 2 contained *S. phureja* mRNAs with either no or little representation in the water-inoculated roots of the susceptible genotype and large presentation in the water inoculated roots of the resistant variety (17 genes). Finally, Group 3 contained mRNAs represented in both resistant and susceptible accessions but several times more abundant in the resistant genotype (11 genes) (Additional file 8).

## Discussion

The identification of new major resistance loci in populations of potato and closely related wild species is an important step of breeding. R loci mapping is commonly performed by phenotyping segregating populations and genotyping them with a large number of genetic markers. However, the identification of R genes is frequently hampered by their nature. It was detected that complex clusters of the NBS-LRR genes in plant genomes are rapidly evolving; plant varieties are commonly characterized by both high levels of copy number variation and disproportionately large SNP accumulation in these genes [5, 19].

Recent development of NGS (next-generation sequencing) techniques has resulted in the accumulation of genomic nucleotide sequences and has provided new opportunities in this field. Resequencing of the genomes of resistant plant genotypes facilitates the identification of R genes of interest (e.g., [4–6]). Potential NBS-LRR genes may also be predicted in genomes with the aid of bioinformatic tools (e.g., [21–23]), and application of these tools for genome analysis may provide large lists of potential RGAs [10].

Despite the application of various NGS-based approaches providing a wide range of new opportunities, the identification of R genes of interest is a complex and time-consuming process. It is likely that a combination of comparative analysis of tissue-specific transcriptomes of susceptible and resistant plant genotypes with bioinformatic predictions of potential NBS-LRR-encoding genes may provide a rapid way to compile a list of candidate RGAs for the genotyping of segregating populations. Since most pathogens commonly infect specific tissues, transcriptome analysis skips both non-specific functional R genes and non-transcribed pseudogenes annotated in the reference genomes. In turn, prediction of NBS-LRR-encoding mRNAs may substantially improve the annotation of related genes in the nucleotide sequence databanks.

To test this approach, we selected two different accessions of *S. phureja* from the VIR collection that likely bear various sets of functional R genes. It was demonstrated previously that these accessions were contrasted in their resistance to the important pathogen *G. rostochiensis* (pathotype Ro1) [25], and we evaluated the number of differentially expressed NBS-LRR genes in their root transcriptomes.

### GPCN infestation and major resistance genes

The penetration of roots by juveniles of root-knot nematodes and their migration to the vascular bundle to arrange a feeding site were similar during both compatible and incompatible interactions (Fig. 1). It is likely that specific nematode recognition occurs after the nematodes inject their esophageal gland secretions to initiate the formation of the feeding site. If the interaction is incompatible, the hypersensitive response can occur as early as 24 h after inoculation and can be identified by a zone of cell death cutting nematode juveniles from the nutrient supply [55]. However, resistance can also be initiated later. *G. rostochiensis* or *G. pallida* can establish the syncytium and become sedentary in resistant tomato and potato plants bearing NBS-LRR resistance genes (*Hero* and *Gpa2*, respectively), but surrounding plant cells further become necrotic, which prevents the completion of the nematode lifecycle. Delayed HR may result from either weak recognition or the late appearance of a nematode effector [55]. Another important feature of nematode inoculation is significant tissue damage resulting in a non-specific wounding stress response induced by plant cell wall fragments. This non-specific wounding response may overlap with the specific response to GPCN or be an integral part of it [56].

A number of quantitative trait loci derived from other cultivated species or their wild relatives were previously identified in Solanaceae with partial resistance to potato cyst nematodes [57]. Several major genes suitable for potato breeding were found. The *H1* locus confers hypersensitive resistance to GPCN (pathotypes Ro1 and Ro4) and was exploited in breeding very actively [33]. *Gro1–4* is a member of the Gro1 locus, which confers nearly absolute resistance to all pathotypes of *G. rostochiensis*, and it is therefore considered a useful resistance gene [58]. Broad-spectrum resistance to *G. rostochiensis* and *G. pallida* is conferred by the *Grp1* gene [59]. Only two *G. rostochiensis* resistance genes were characterized at the molecular level: *Gpa2* from *S. tuberosum* ssp. *andigena* [60] and *Gro1–4* from *S. spegazzinii* [58]. *Gpa2* and *Gro1–4* genes and a resistance gene from tomato (*Hero*) belong to the NBS-LRR family.

### *S. phureja* model

It was known that accession i-0144786 was susceptible, whereas i-0144787 was highly resistant to GPCN [25], and these degrees of resistance were confirmed in the present research (Figs. 1 and 2; Table 1). It may be assumed that the root transcriptome of i-0144787 plants contains mRNAs coding for NBS-LRR genes that are not transcribed (or transcribed at significantly lower levels) in the roots of i-0144786 plants. To test this hypothesis, the root transcriptomes of resistant and susceptible genotypes collected 72 h after inoculation with either *G. rostochiensis* or water were sequenced. The lists of DEGs were compiled, and GO term analysis revealed the enrichment of house-keeping genes in down-regulated groups and stress-related genes in up-regulated groups (Table 3; Additional file 2). This result reflects the typical response to either tissue wounding alone (inoculation with water) or a combination of tissue wounding and nematode infestation (inoculation with GPCN). It was also found that only one corresponding gene in the reference *S. tuberosum* genome was annotated as belonging to the NBS-LRR family, which complicates the selection of candidate R genes for further analysis. This finding likely resulted from a stringent significance threshold of a standard pipeline and a scarce annotation. Thus, we used additional information on 755 NB-LRR loci predicted in the *S. tuberosum* genome [46]. This information revealed approximately 300 transcripts in the root transcriptomes of *S. phureja* genotypes potentially encoding NBS-LRR-related proteins.

Interestingly, genotypes i-0144786 and i-0144787 were characterized by different subsets of expressed NBS-LRR-like RGAs (Additional file 8). These accessions evolved under different conditions and pathogenic pressure. In our opinion, systemic comparison between the differential expression of RGA subsets with phenotypic screening of the resistance to various pathogens may be considered a prospective source for the identification of new candidate R genes. In this study, four transcriptomes were compared (roots of resistant and susceptible genotypes taken 72 h after inoculation with either water or nematodes). The nematode juveniles significantly damage tissues during the penetration process, resulting in non-specific wounding stress. The procedure of inoculation itself also damages root tissues. Thus, the transcriptome of water-inoculated roots may be considered an appropriate control to reveal the biotic stress response components. To select the potential R genes, the NBS-LRR-like genes were divided into three groups (Additional file 8). The first group contained two transcripts of *S. phureja* genes present in the roots of the resistant genotype i-0144787 and either absent or present in a very small amount in the susceptible i-0144786. The second group contained 17 transcripts absent or present in small amounts in the root transcriptome of water-inoculated susceptible plants. Strong resistance to *G. rostochiensis* is commonly based on the rapid hypersensitive response followed by the programmed cell death of the neighboring plant cells, and it is likely that efficient NBS-LRR genes should be expressed before nematode infestation (e.g., [12, 14]). We hypothesized that low expression of NBS-LRR receptor genes in the absence of GPCN infestation results in a delay of the hypersensitive response and may provide time for successful nematode progression. Finally, the third group includes transcripts present in the roots of both genotypes but considerably more abundant in the i-0144787 accession.

## Conclusion and perspectives

Comparative analysis of the root transcriptomes of *Solanum phureja* genotypes with additional computational predictions of mRNAs coding for NBS-LRR-like proteins revealed a reasonable number of candidate R genes for further co-segregation analysis. In our opinion, this approach provides a rapid method of candidate gene selection and may be used in parallel with more sophisticated studies based on genome resequencing. If successful, this approach considerably accelerates the time it takes to identify resistance genes for targeted breeding. It should also be mentioned that in addition to the source of new genes, *S. phureja* is the donor of fertile-type cytoplasm, which is very promising for the genetic improvement of the common potato *S. tuberosum* [61].

## Additional files

Additional file 1: Positions of potential resistance genes (Jupe et al. [46]) in the reference *S. tuberosum* genome. (XLSX 44 kb)

Additional file 2: Comparison of root transcriptomes of resistant and susceptible *S. phureja* genotypes collected 72 h after inoculation with either nematode or water. (XLSX 236 kb)

Additional file 3: The log_2_(FC) values and experimental fold changes for DEGs verified with qRT-PCR as well as the primer sequences constructed and used in qRT-PCR. (XLSX 13 kb)

Additional file 4: qRT-PCR validation of DEGs (relative mRNA levels of 10 genes obtained using gene-specific primers and cDNA of susceptible and resistant *S. phureja* genotypes (24H = i-0144786 and 36H = i-0144787, respectively)). (PDF 17 kb)

Additional file 5: GO terms enriched for down-regulated transcripts in the roots of the nematode-resistant *S. phureja* genotype. (PDF 399 kb)

Additional file 6: GO terms enriched for up-regulated transcripts in the roots of the nematode-resistant *S. phureja* genotype. (PDF 240 kb)

Additional file 7: *S. phureja* mRNAs corresponding to resistance genes predicted in the reference *S. tuberosum* genome (Jupe et al. [46]). (XLSX 62 kb)

Additional file 8: List of candidate *S. phureja* NBS-LRR-encoding genes providing resistance to *G. rostochiensis. (XLSX 18 kb)*
