# Supplementary figures and images for: Differential expression of NBS-LRR-encoding genes in the root transcriptomes of two Solanum phureja genotypes with contrasting resistance to Globodera rostochiensis
Source: BMC Plant Biol. 2017 Dec 28;17(Suppl 2):251. doi: 10.1186/s12870-017-1193-1 (PMC5751396; doi:10.1186/s12870-017-1193-1)

PGSC0003DMG400004561

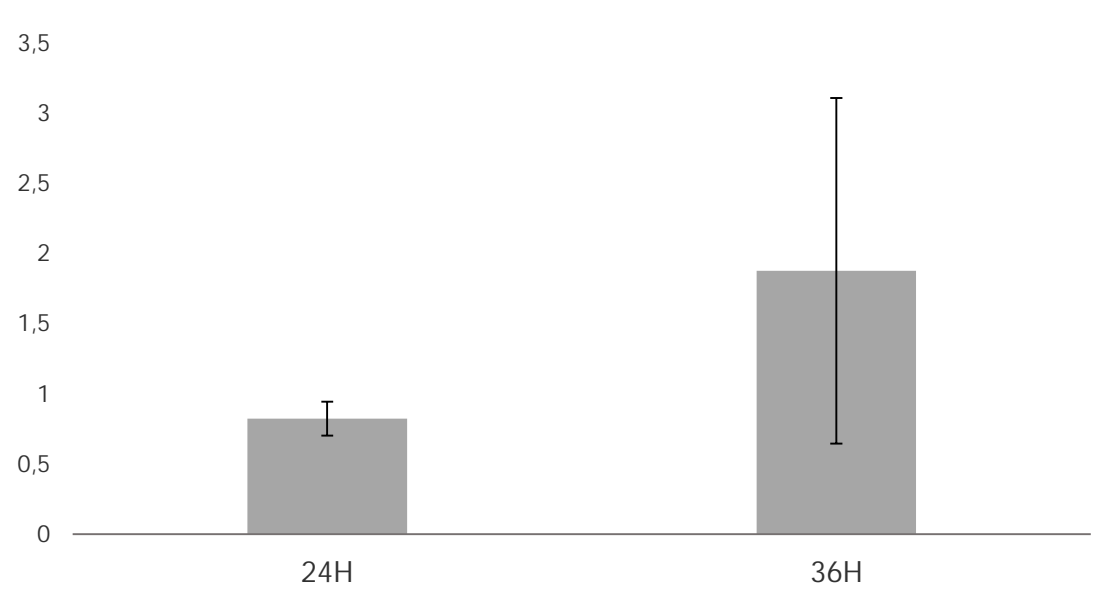

PGSC0003DMG400005970\*

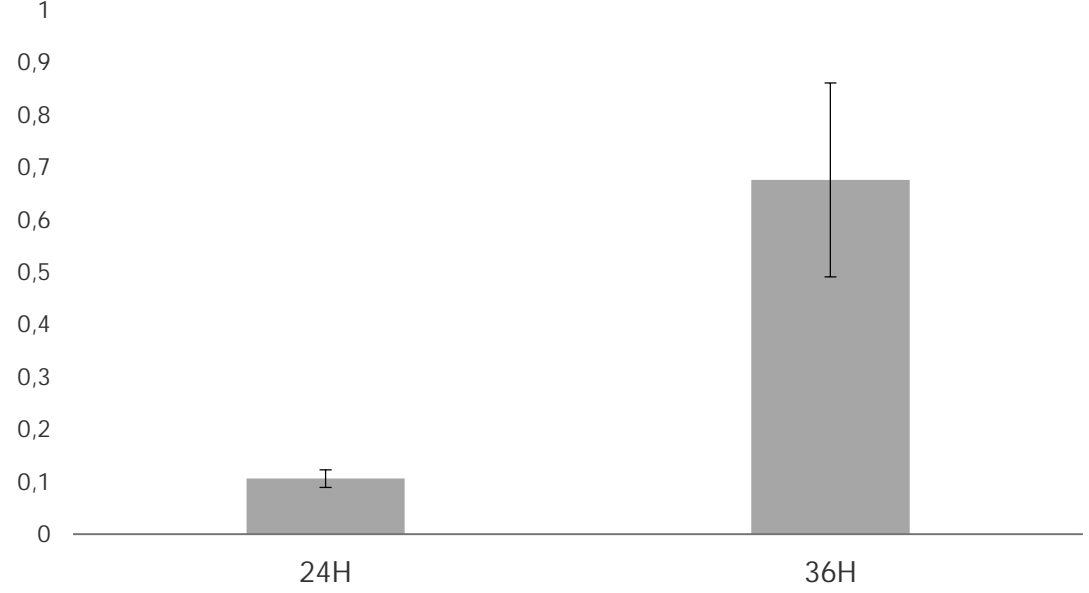

PGSC0003DMG400011517\*

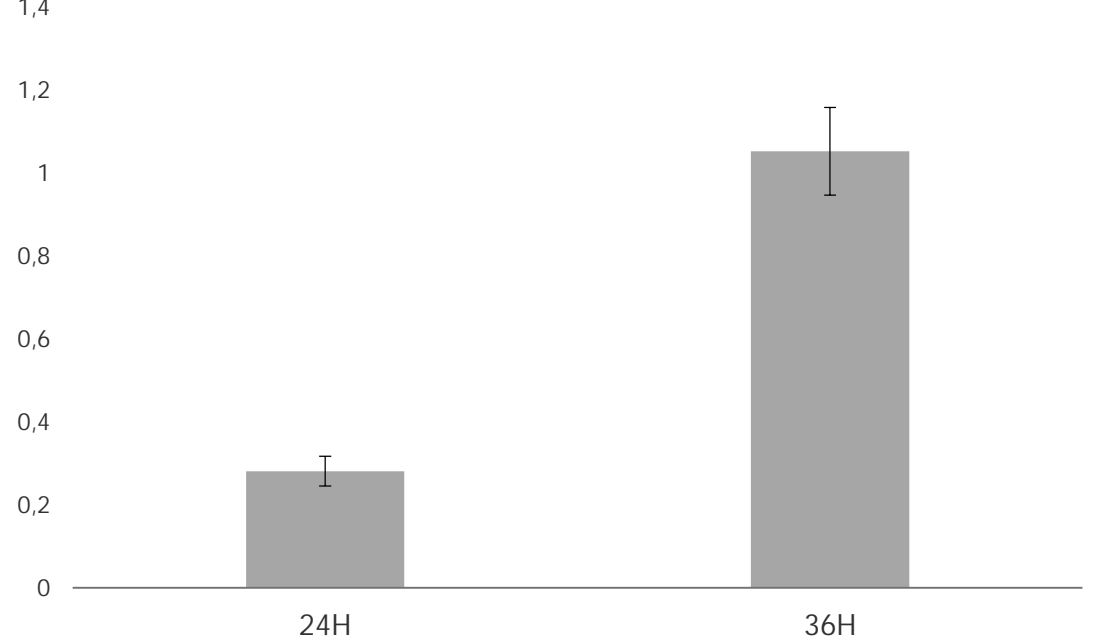

PGSC0003DMG400013308\*

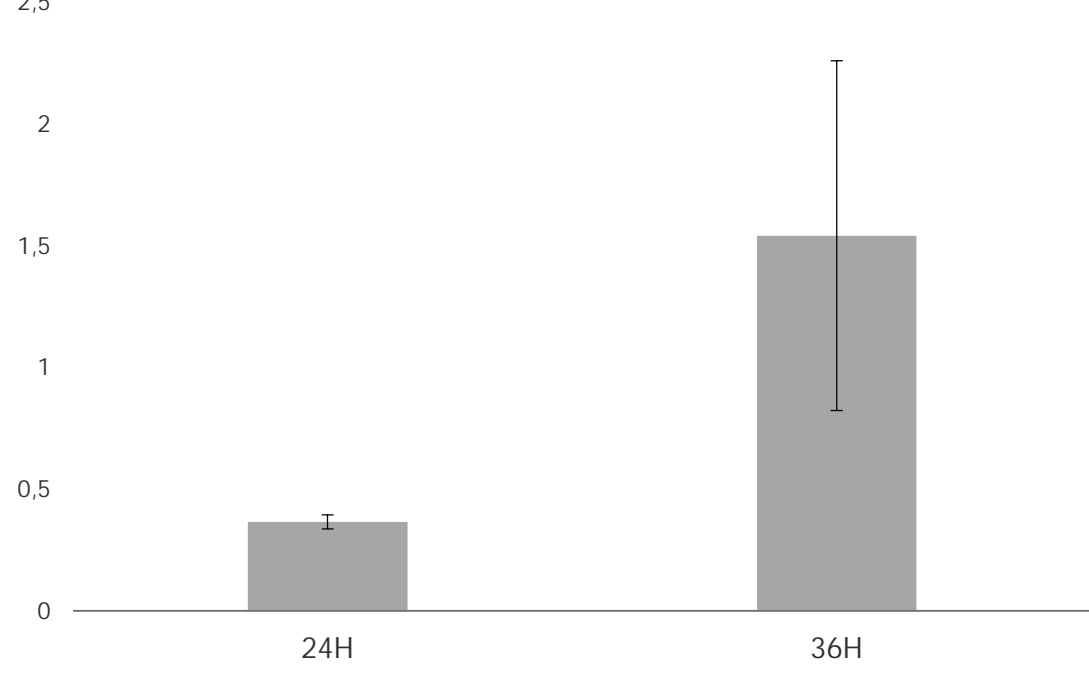

PGSC0003DMG400018464\*

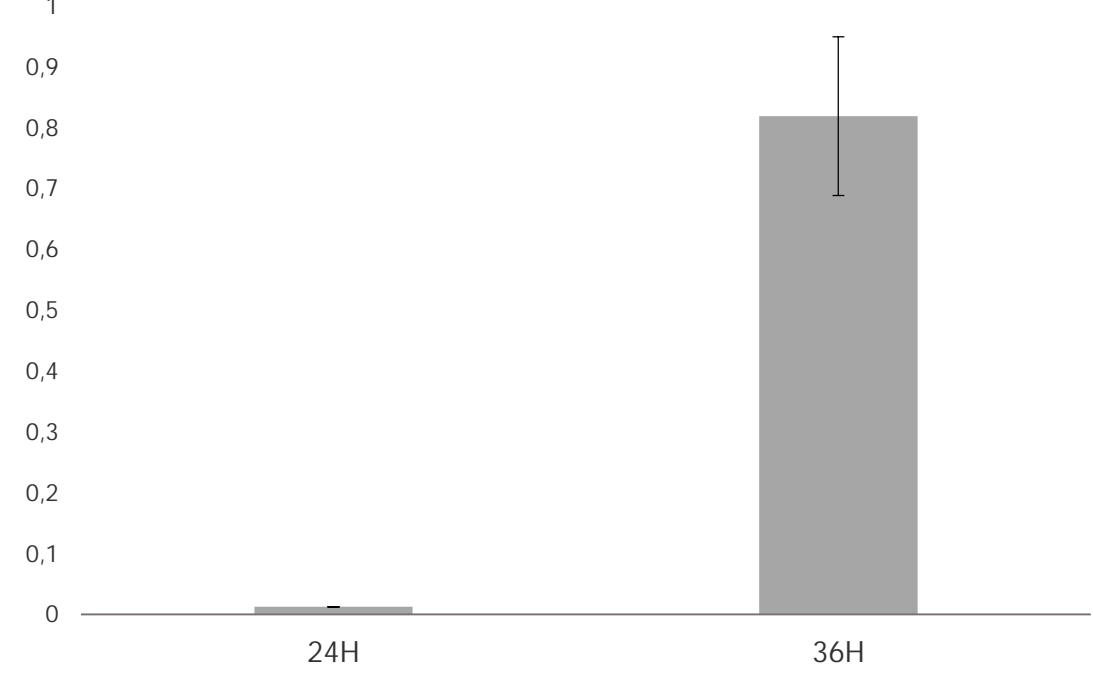

PGSC0003DMG400020722\*

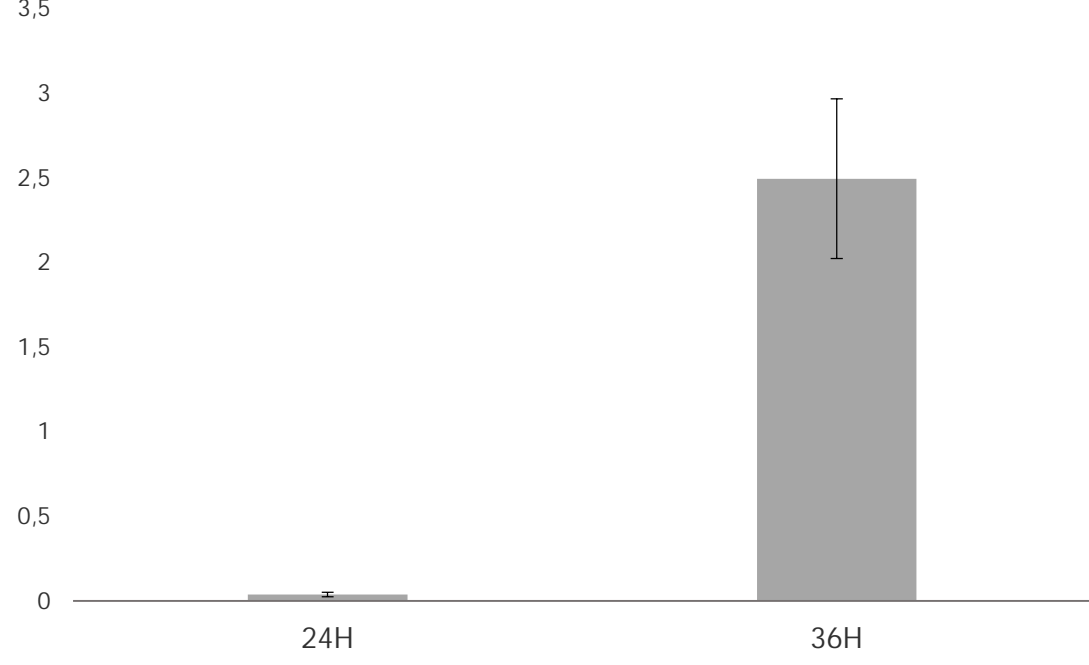

PGSC0003DMG400023288\*

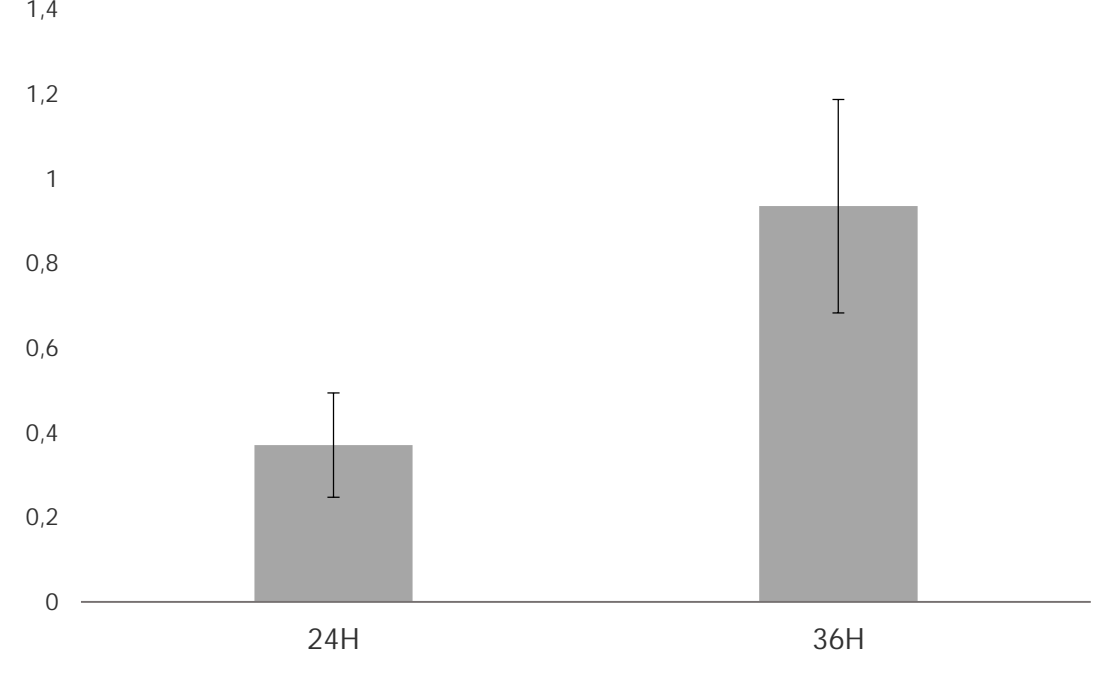

PGSC0003DMG400026666\*

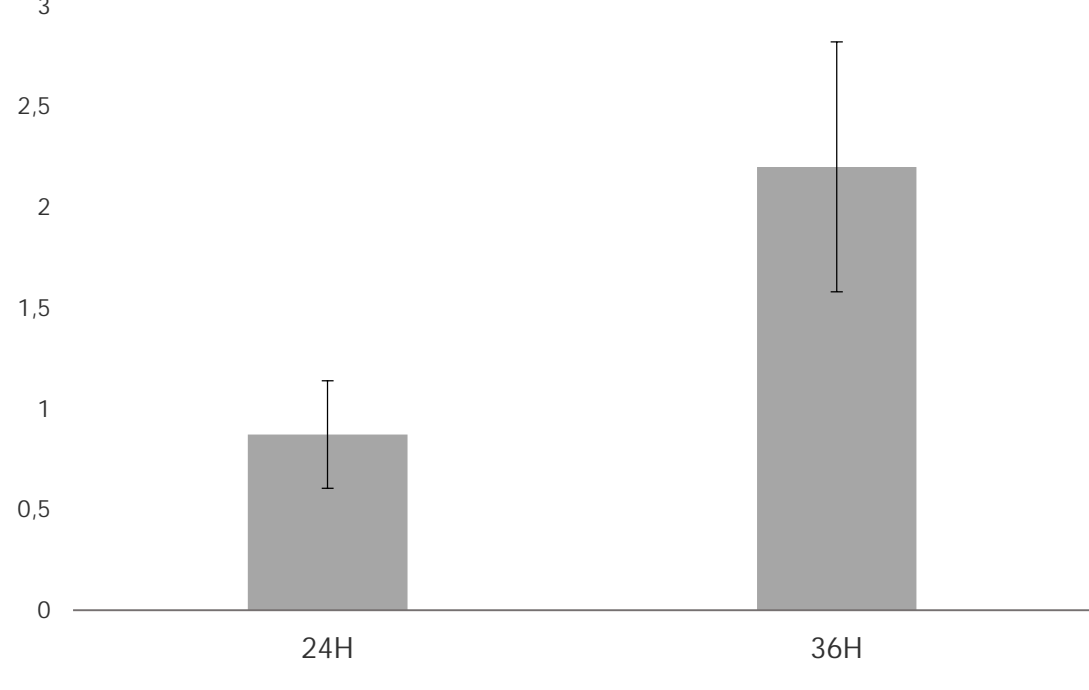

PGSC0003DMG402016602\*

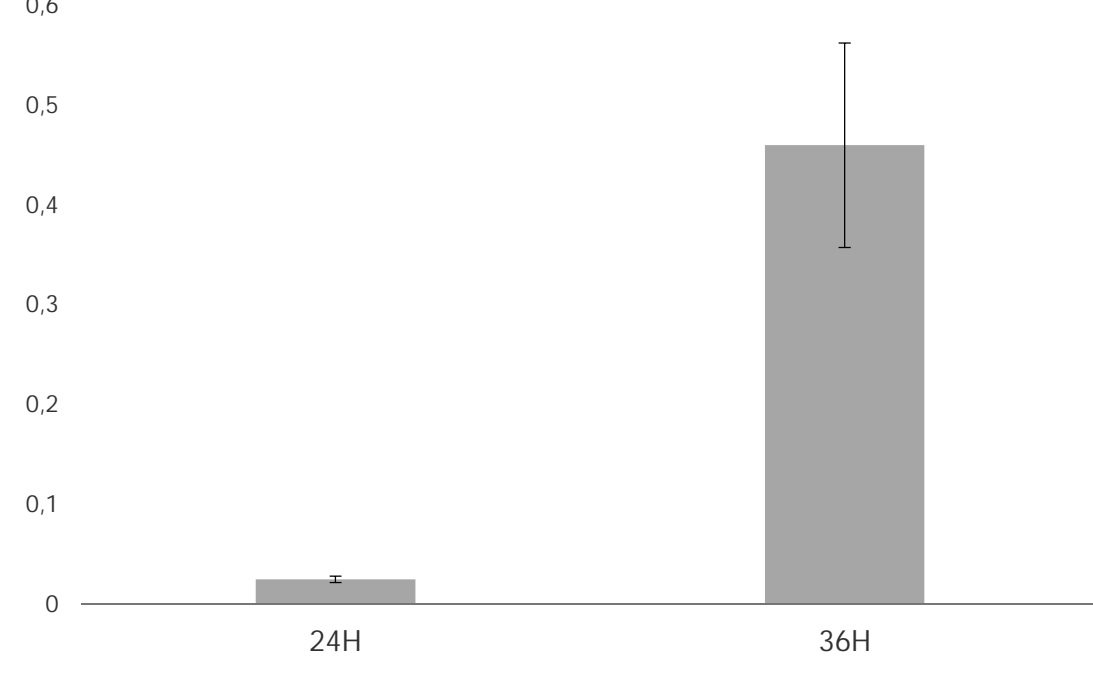

PGSC0003DMG402027402

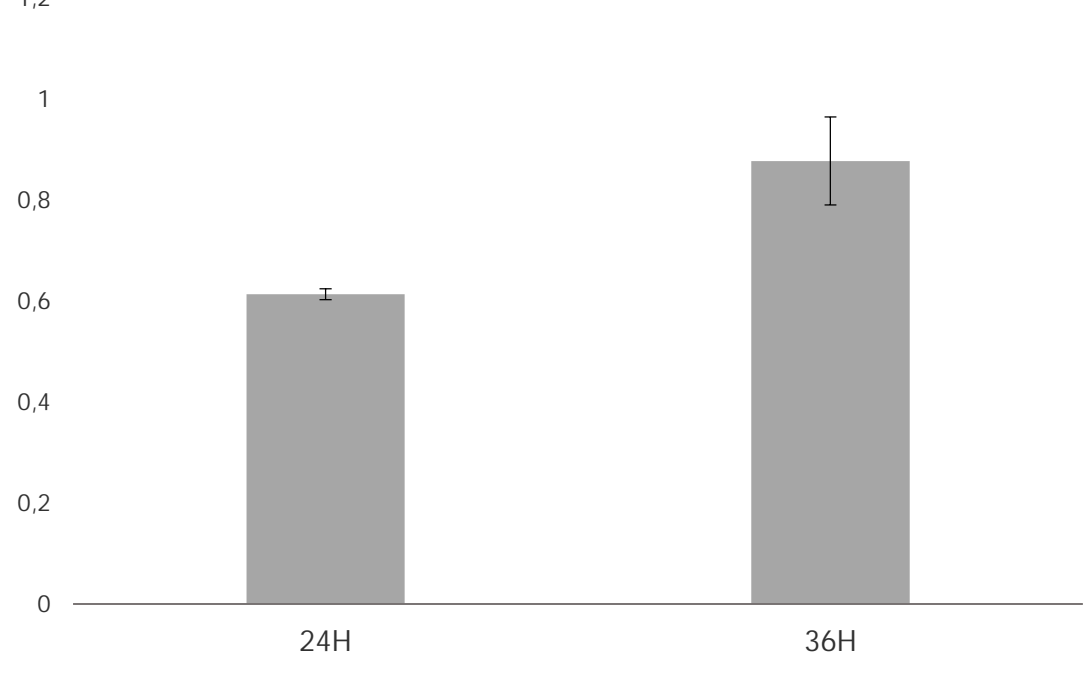

Supplement: Supplementary file 4 — qRT-PCR validation of DEGs (relative mRNA levels of 10 genes obtained using gene-specific primers and cDNA of susceptible and resistant S. phureja genotypes (24H = i-0144786 and 36H = i-0144787, respectively)). (PDF 17 kb) [file 12870_2017_1193_MOESM4_ESM.pdf]

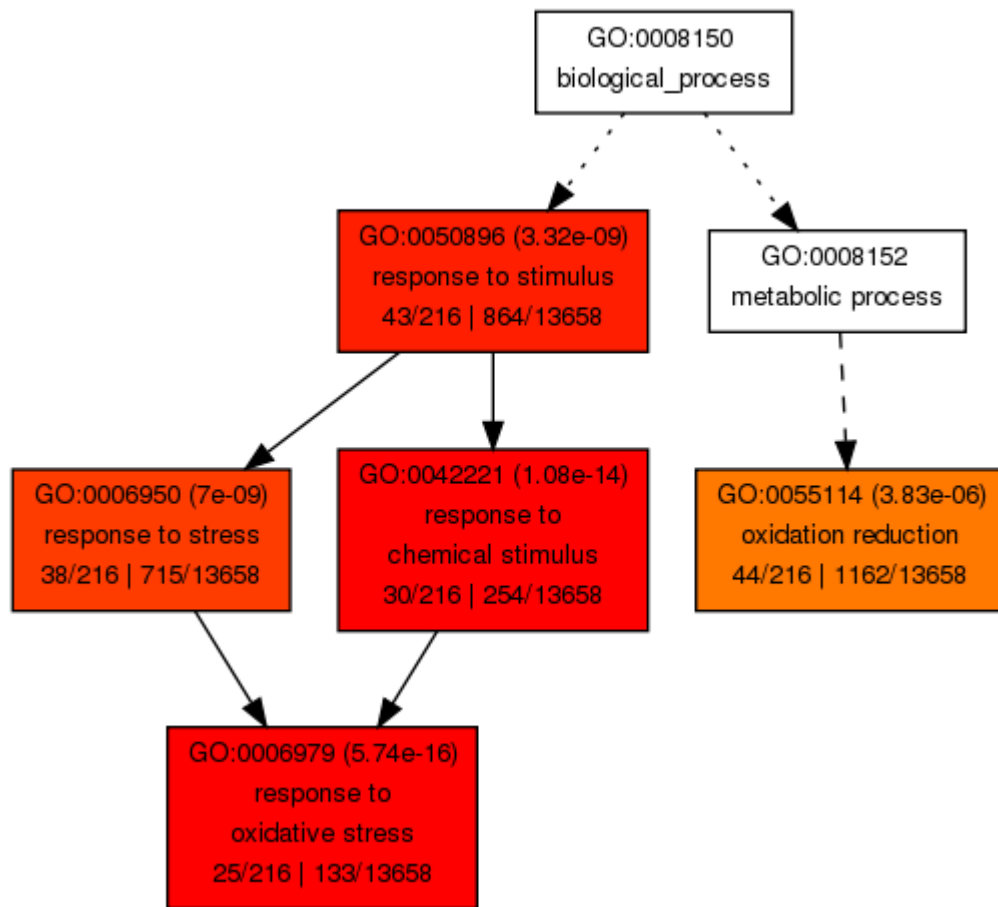

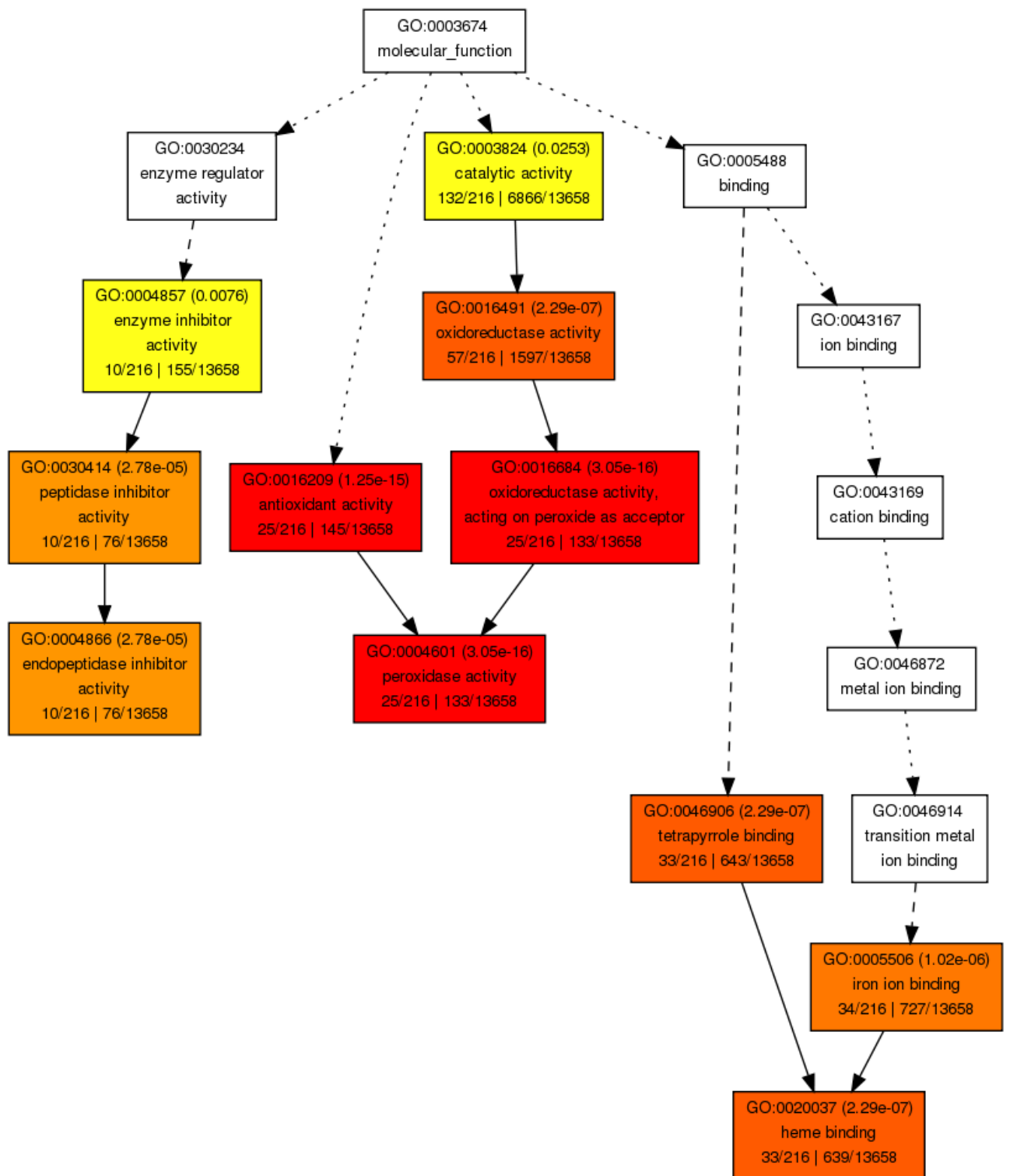

Supplement: Supplementary file 6 — GO terms enriched for up-regulated transcripts in the roots of the nematode-resistant S. phureja genotype. (PDF 240 kb) [file 12870_2017_1193_MOESM6_ESM.pdf]
