# Supplementary material for: Differential expression of NBS-LRR-encoding genes in the root transcriptomes of two Solanum phureja genotypes with contrasting resistance to Globodera rostochiensis
Source: BMC Plant Biol. 2017 Dec 28;17(Suppl 2):251. doi: 10.1186/s12870-017-1193-1 (PMC5751396; doi:10.1186/s12870-017-1193-1)
Supplement: Supplementary file 5 — GO terms enriched for down-regulated transcripts in the roots of the nematode-resistant S. phureja genotype. (PDF 399 kb) [file 12870_2017_1193_MOESM5_ESM.pdf]

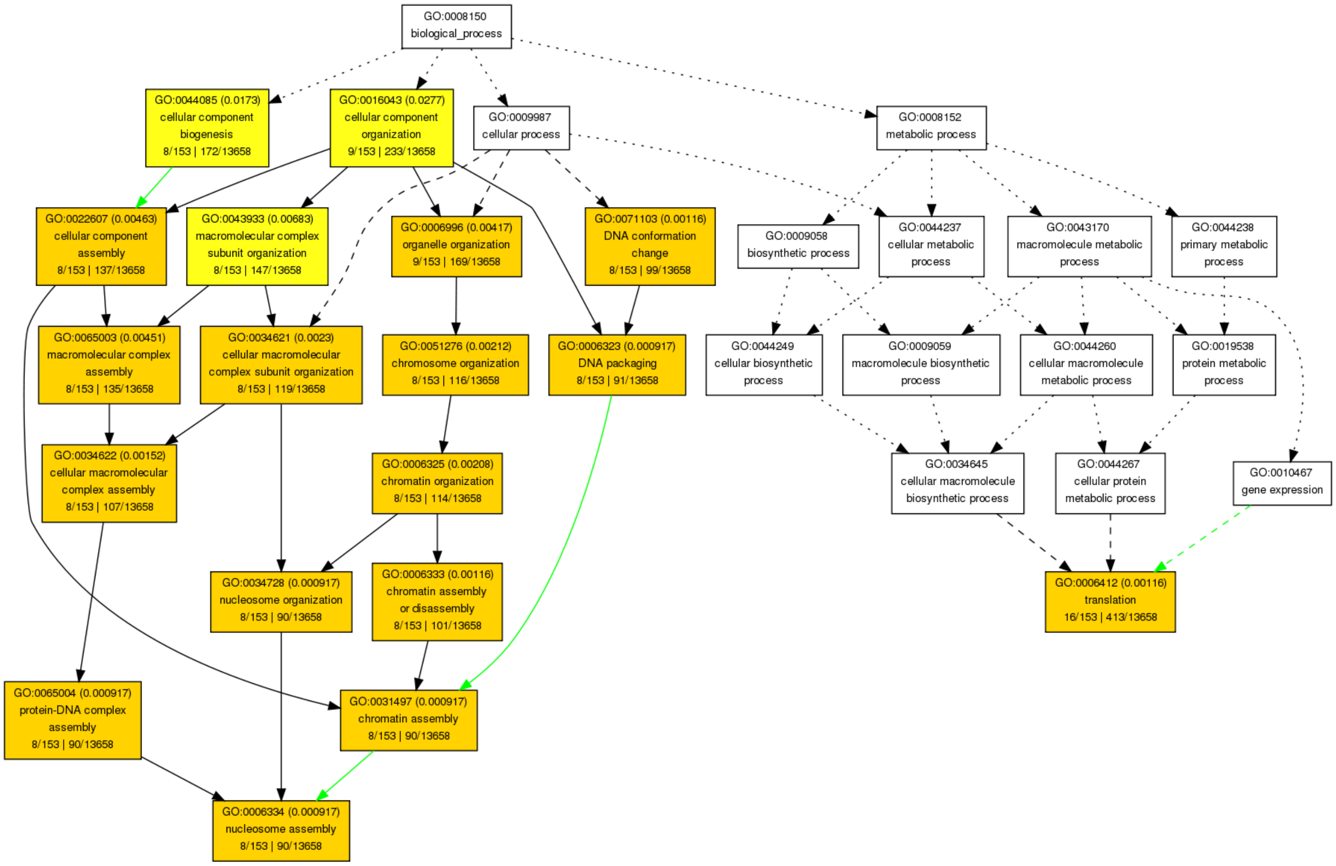
GO:0008150
biological_process
GO:0044085 (0.0173)
cellular component
biogenesis
8/153 | 172/13658
GO:0016043 (0.0277)
cellular component
organization
9/153 | 233/13658
GO:0009987
cellular process
GO:0008152
metabolic process
GO:0022607 (0.00463)
cellular component
assembly
8/153 | 137/13658
GO:0043933 (0.00683)
macromolecular complex
subunit organization
8/153 | 147/13658
GO:0006996 (0.00417)
organelle organization
9/153 | 169/13658
GO:0071103 (0.00116)
DNA conformation
change
8/153 | 99/13658
GO:0009058
biosynthetic process
GO:0044237
cellular metabolic
process
GO:0043170
macromolecule metabolic
process
GO:0044238
primary metabolic
process
GO:0065003 (0.00451)
macromolecular complex
assembly
8/153 | 135/13658
GO:0034621 (0.0023)
cellular macromolecular
complex subunit organization
8/153 | 119/13658
GO:0051276 (0.00212)
chromosome organization
8/153 | 116/13658
GO:0006323 (0.000917)
DNA packaging
8/153 | 91/13658
GO:0044249
cellular biosynthetic
process
GO:0009059
macromolecule biosynthetic
process
GO:0044260
cellular macromolecule
metabolic process
GO:0019538
protein metabolic
process
GO:0034622 (0.00152)
cellular macromolecular
complex assembly
8/153 | 107/13658
GO:0006325 (0.00208)
chromatin organization
8/153 | 114/13658
GO:0034645
cellular macromolecule
biosynthetic process
GO:0044267
cellular protein
metabolic process
GO:0010467
gene expression
GO:0034728 (0.000917)
nucleosome organization
8/153 | 90/13658
GO:0006333 (0.00116)
chromatin assembly
or disassembly
8/153 | 101/13658
GO:0006412 (0.00116)
translation
16/153 | 413/13658
GO:0065004 (0.000917)
protein-DNA complex
assembly
8/153 | 90/13658
GO:0031497 (0.000917)
chromatin assembly
8/153 | 90/13658
GO:0006334 (0.000917)
nucleosome assembly
8/153 | 90/13658

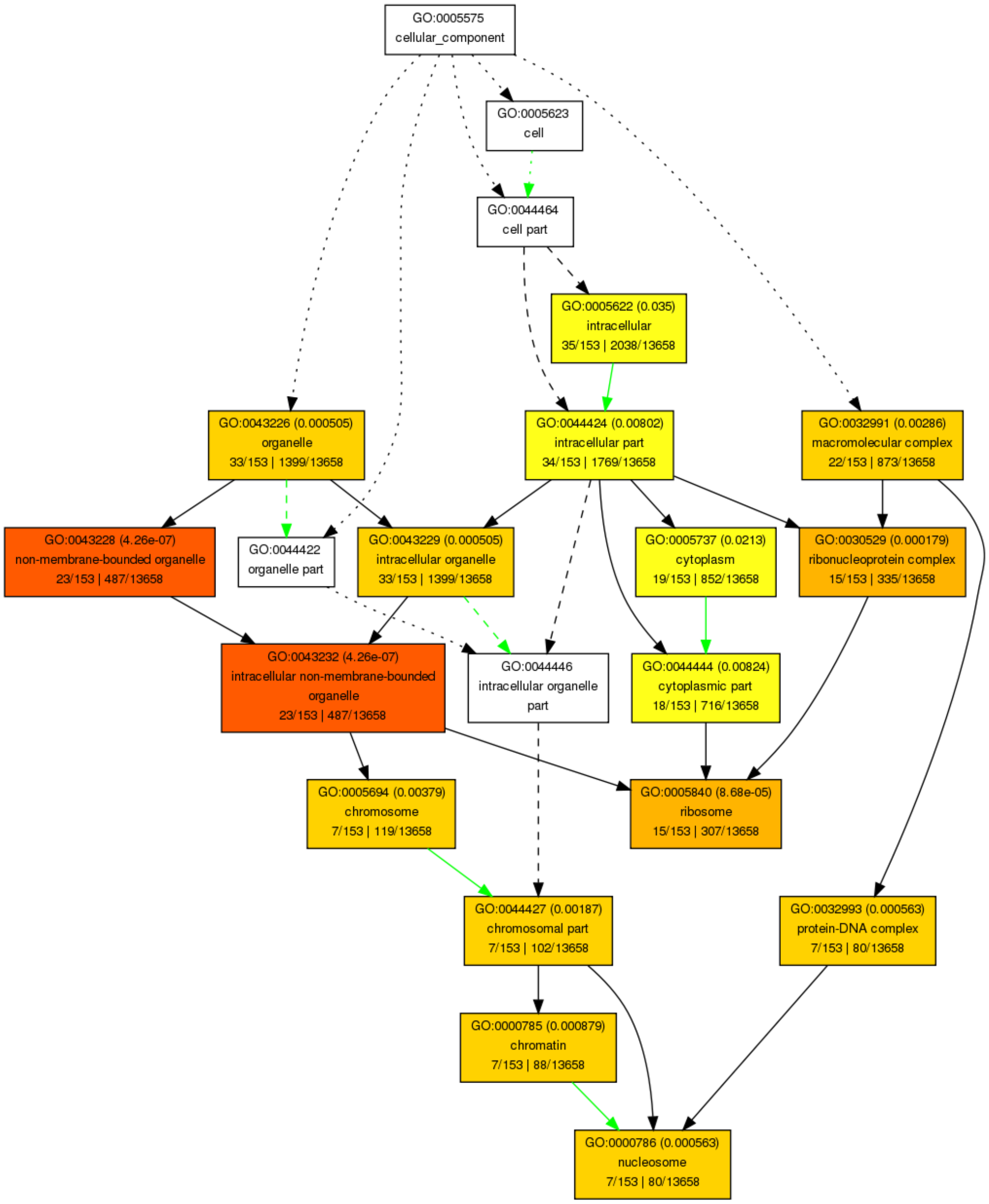
GO:0005575
cellular_component
GO:0005623
cell
GO:0044464
cell part
GO:0005622 (0.035)
intracellular
35/153 | 2038/13658
GO:0043226 (0.000505)
organelle
33/153 | 1399/13658
GO:0044424 (0.00802)
intracellular part
34/153 | 1769/13658
GO:0032991 (0.00286)
macromolecular complex
22/153 | 873/13658
GO:0043228 (4.26e-07)
non-membrane-bounded organelle
23/153 | 487/13658
GO:0044422
organelle part
GO:0043229 (0.000505)
intracellular organelle
33/153 | 1399/13658
GO:0005737 (0.0213)
cytoplasm
19/153 | 852/13658
GO:0030529 (0.000179)
ribonucleoprotein complex
15/153 | 335/13658
GO:0043232 (4.26e-07)
intracellular non-membrane-bounded
organelle
23/153 | 487/13658
GO:0044446
intracellular organelle
part
GO:0044444 (0.00824)
cytoplasmic part
18/153 | 716/13658
GO:0005694 (0.00379)
chromosome
7/153 | 119/13658
GO:0005840 (8.68e-05)
ribosome
15/153 | 307/13658
GO:0044427 (0.00187)
chromosomal part
7/153 | 102/13658
GO:0032993 (0.000563)
protein-DNA complex
7/153 | 80/13658
GO:0000785 (0.000879)
chromatin
7/153 | 88/13658
GO:0000786 (0.000563)
nucleosome
7/153 | 80/13658

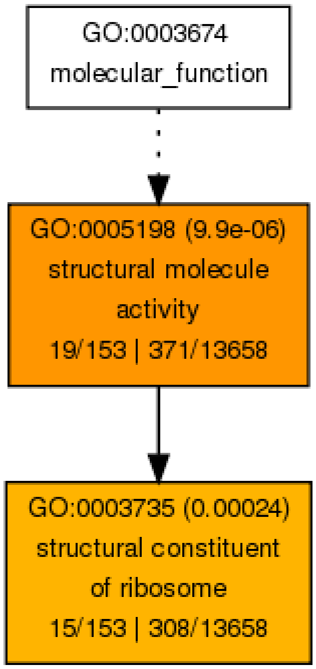
GO:0003674
molecular_function
GO:0005198 (9.9e-06)
structural molecule
activity
19/153 | 371/13658
GO:0003735 (0.00024)
structural constituent
of ribosome
15/153 | 308/13658
